# Concurrent Terahertz Spin Excitations and Phase Shift Control in Fe_4_Nb_2_O_9_: A Material for Synergizing Computation and Communication Technologies

**DOI:** 10.1002/advs.75555

**Published:** 2026-05-13

**Authors:** Brijesh Singh Mehra, Karan Datt Sharma, Sanjeev Kumar, Gaurav Dubey, Mitanshi Gupta, Ravi Shankar Singh, Dibakar Roy Chowdhury, Christine Martin, Antoine Maignan, Kiran Singh, Dhanvir Singh Rana

**Affiliations:** ^1^ Department of Physics Indian Institute of Science Education and Research Bhopal Bhopal Madhya Pradesh India; ^2^ School of Engineering Anurag University Ghatkesar Telangana India; ^3^ CRISMAT Laboratoire de Cristallographie et Sciences des Matériaux ENSICAEN UNICAEN CNRS Normandie Université Caen France; ^4^ Department of Physics Dr. B. R. Ambedkar National Institute of Technology Jalandhar Punjab India

**Keywords:** magnetism, magnetoelastic, magnons, phase shifter, terahertz spectroscopy

## Abstract

Driven by escalating demands from emerging applications and inherent limitations of current technologies, terahertz (THz) science has surged as a cornerstone for 6G+ communication systems and magnonic computation. Phase shifters, essential for THz wave manipulation, drive communication advances, while magnons enable ultrafast, energy‐efficient processing. Ideally, a multifunctional material co‐hosting both would integrate these fields towards lab‐on‐a‐chip applications; yet, no such experimental realization exists. Herein, a novel platform is presented to integrate intrinsic THz phase shifting and spin excitations within a single material system. Fe_4_Nb_2_O_9_, selected as a model compound, demonstrates a remarkable intrinsic phase‐shift of 304.7° at around 0.5 THz in the temperature range of 30–93 K. This gigantic effect either matches or surpasses the existing metasurface‐based free‐space THz phase shifters − with a correlated phase‐frequency relationship across a wide sub‐THz bandwidth (0.1 to 0.56 THz). The experimental results, corroborated by theoretical lattice vibration calculations, attribute the observed THz phase‐shift to dielectric modulation in Fe_4_Nb_2_O_9_. Complementing these findings, two distinct THz magnon excitations were detected above 0.56 THz in its antiferromagnetic phase. This unique coexistence of phase modulation and spin dynamics positions Fe_4_Nb_2_O_9_ as a paradigmatic multifunctional material‐a model compound for next‐generation miniaturized THz communication and computation platforms.

## Graphical abstract

A multifunctional terahertz (THz) platform is demonstrated, in a model material Fe_4_Nb_2_O_9_, exhibiting a large intrinsic THz phase shift alongside distinct THz magnon excitations in its antiferromagnetic phase. This coexistence of phase modulation and spin dynamics offers a promising pathway toward integrated THz communication and magnonic computation within a single material platform, while also inspiring the discovery of next‐generation multifunctional THz quantum materials.

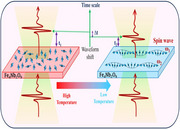

## 1 Introduction

The accelerating demand for high‐speed data transmission and secure information processing is increasingly straining conventional technologies, which are limited by finite spectral bandwidth and the scaling constraints of charge‐based electronic circuits commonly associated with Moore's law [1, 2, 3]. As a result, computation is moving beyond traditional electronics toward alternative paradigms such as spin‐wave‐based (magnonic) technologies, where information is encoded in collective spin excitations that can be manipulated on ultrafast timescales with low energy dissipation [4, 5, 6, 7]. In parallel, wireless communication has progressed from 2G to 5G, boosting data rates from kilobits to multi‐gigabits per second has improved connectivity tremendously but exposed an imminent bandwidth bottleneck [8, 9]. Terahertz (THz) electromagnetic spectrum (0.1–10 THz) offers a transformative frontier where both computation and communication technologies can overlap and advance significantly. It provides orders‐of‐magnitude larger spectral bandwidth for wireless links while matching the intrinsic energy scale of antiferromagnetic spin excitations relevant for next‐generation magnonic functionality [10, 11]. For the emerging applications, such as augmented reality, autonomous systems, and the Internet of Things, combined with the physical limits of existing technologies, render the THz frequency band indispensable for future advancements in high‐speed communication and computation.

Crucial device development for THz‐based communication requires the development of functional devices such as switches, filters, mixers, polarizers, and especially modulators like phase shifters for beam‐steering, signal synchronization, and interference mitigation of THz light [12, 13, 14, 15, 16, 17, 18, 19, 20, 21]. Existing THz phase shifters rely on materials that modulate through changes in carrier density (semiconductors, 2D materials) or molecular rotation in liquid crystals. These materials provide only limited phase shifts, typically enhanced by integrating with metasurfaces that improve phase control but bring limitations of narrow bandwidths and cumbersome fabrication [22, 23, 24, 25, 26, 27, 28, 29]. A promising path forward is focusing on intrinsic THz phase shift mechanisms by identifying materials that serve as intrinsic, tunable dielectric modulators [30, 31, 32]. Magneto‐elastic mechanisms in such THz phase shifters have recently shown potential for enhancing intrinsic phase shifts [30, 31], although this remains underexplored. Complementary to this, a critical component for spin‐wave‐based computation is the identification of magnons‐the quanta of spin waves‐in a magnetic system. The energy of these magnons is fundamentally governed by the type of magnetic ordering, magnetocrystalline anisotropy, and the strength of inter‐spin exchange interactions. Consequently, magnons in ferromagnets typically resonate at gigahertz frequencies, whereas those in antiferromagnets access the THz regime. The latter offer compelling advantages, including ultrafast dynamics (THz speeds), extended propagation lengths, and minimal ohmic power dissipation, making antiferromagnetic magnons ideal for high‐speed THz information processing [33, 34, 35, 36, 37, 38, 39]. In this context, it is in principle possible for a system to simultaneously exhibit phase shifting and spin‐wave excitation properties, which would potentially synergize THz communication and computation fields. Yet, no experimentally known system currently unites these functionalities intrinsically.

Therefore, we introduce a novel THz‐active multifunctional material, Fe_4_Nb_2_O_9_, distinguished by two key properties in the THz frequency range. First, it exhibits a remarkable intrinsic THz phase shift of 304.7° between 30 and 93 K at 0.56 THz, with a correlated phase‐frequency relationship spanning a broad range from 0.1 to 0.56 THz. Second, at 8 K, two distinct magnon excitations are observed at 0.62 and 0.91 THz. The coexistence of a large intrinsic THz phase shift and the presence of magnons underscores the significant potential of Fe_4_Nb_2_O_9_ as a compact multifunctional platform for future THz communication and computation applications, enabling integrated control of phase modulation and spin‐wave‐based information processing.

## 2 Results and Discussion

**FIGURE 1:**
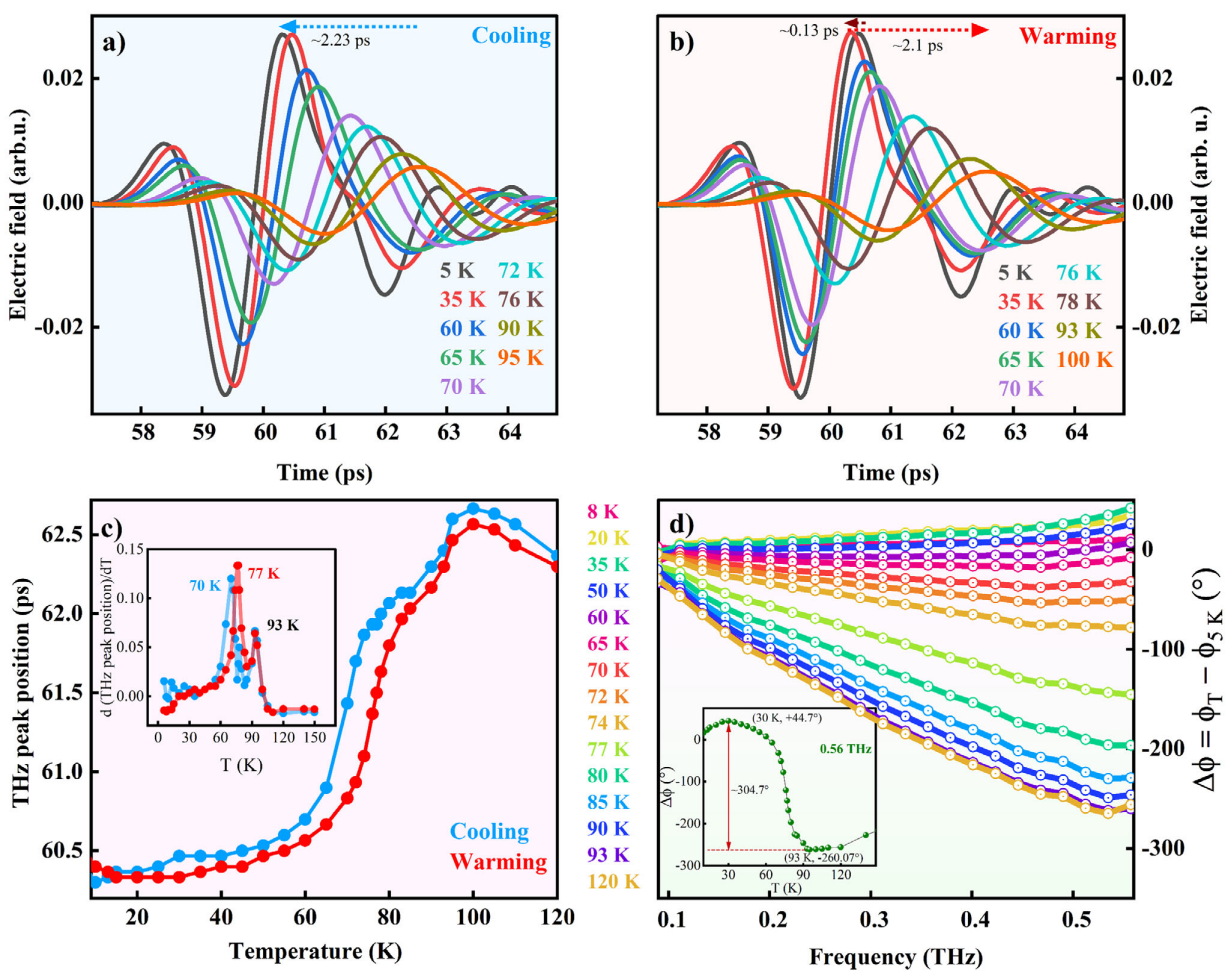
Transmitted THz response as it passes through the sample. THz waveform in (a) cooling cycle, and (b) warming cycle. (c) THz peak position as a function of temperature in warming and cooling cycles. Inset shows its derivative as a function of temperature, revealing two transitions at 93 and 77 (70) K in a warming (cooling) cycle. (d) THz phase shift with respect to 5 K (Δϕ) as a function of frequency. Inset shows the THz phase shift with respect to 5 K vs. temperature at 0.56 THz.

Figure 1a–c depicts the THz transmission observed as the incident THz radiation passes through Fe_4_Nb_2_O_9_. As the temperature decreases, the THz waveform undergoes an enormous shift in time by 2.23 ps from ∼95 to 5 K (Figure 1a). Conversely, upon increasing the temperature, a bidirectional THz waveform shift is observed, first by 0.13 ps between 5 and 35 K and then by 2.1 ps from 35 to 100 K, as shown in Figure 1b. (Figure 1c) This distinct change in behavior is more clearly emphasized in the peak position plot as a function of temperature during both the warming and cooling cycles, which depicts a thermal hysteresis in Fe_4_Nb_2_O_9_ reflecting its complex phase transition characteristics. Its inset shows the temperature derivative of peak position, which highlights two temperatures, 93 and 70 K (77 K) in the cooling (warming) protocol, which corresponds to the magnetic and structural transition of Fe_4_Nb_2_O_9_, respectively [40, 41, 42, 43]. To analyze the THz frequency component response, we performed a Fast Fourier Transform on the THz time‐domain waveform, which provides the corresponding phase and amplitude in the frequency domain. The amplitude in the frequency domain can be divided into two regions: the phase shift region and the magnetic excitations region (Figure S2). The details of the latter will be discussed later. The phase shift with respect to 5 K, as shown in Figure 1d, shows a correlated phase‐frequency relation in the range of 0.10–0.56 THz with a THz phase shift of 260° at 0.56 THz. The maximum phase shift observed at 0.56 THz is ∼304.7° between 30 and 93 K as shown in the inset of Figure 1d. This unprecedentedly large phase shift is only intrinsic in nature, contrary to the metasurface‐assisted free‐space phase modulators.

**FIGURE 2:**
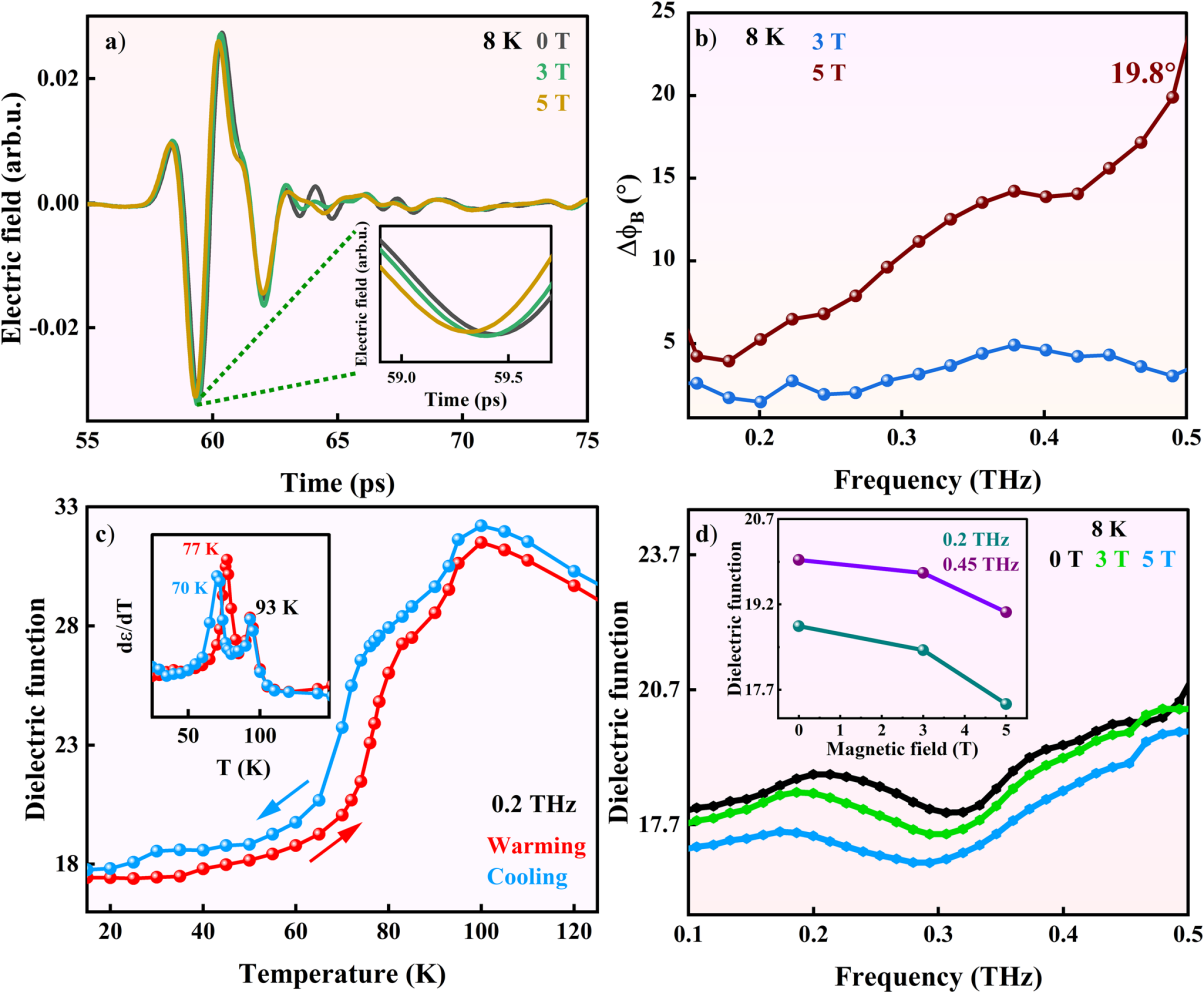
Magnetic field‐dependent THz measurements. (a) THz waveform at 8 K under different applied magnetic fields. (b) Phase shift with respect to 0 T (Δϕ_B_) as a function of frequency at different magnetic fields. (c) Dielectric function and its derivative (inset) as a function of temperature in warming and cooling cycle. (d) Dielectric function vs. frequency at 8 K under different magnetic fields. Inset shows the dielectric function vs magnetic field.

Since Fe_4_Nb_2_O_9_ is a magnetic material, it is essential to understand how the phase shift varies in the presence of an external magnetic field. Figure 2a illustrates the magnetic field dependence of the THz waveforms at 8 K, showcasing the waveform modulation with the magnetic field. Notably, we observe a rare magnetic‐field‐dependent phase shift, reaching a maximum of 19.8° at 0.5 THz (Figure 2b). This behavior suggests a magneto‐electric coupling in Fe_4_Nb_2_O_9_, where the magnetic field influences the THz electric field phase.

**FIGURE 3:**
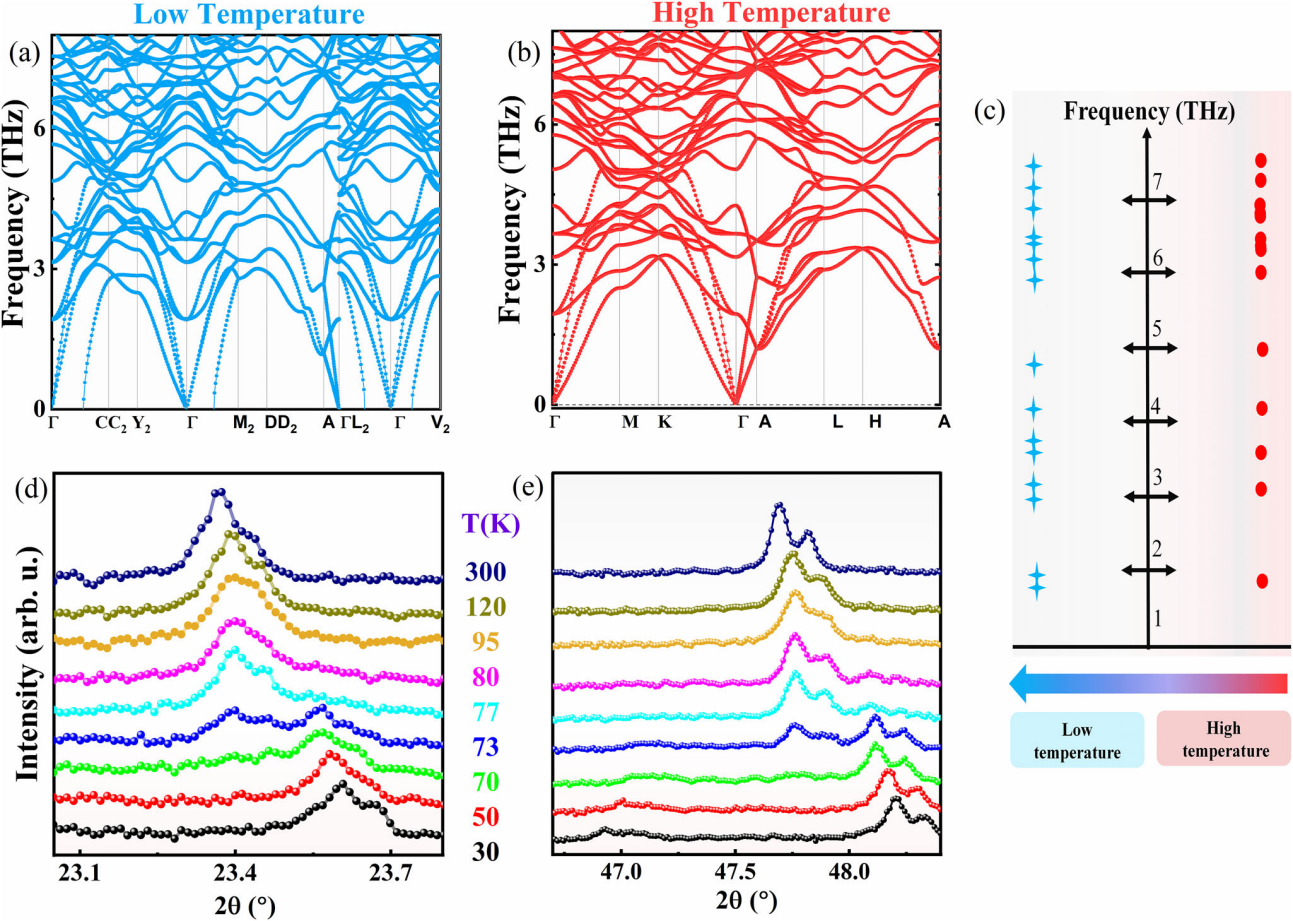
(a, b) Phonon dispersion curves in the low and high temperature phases. (c) Schematic representation of phonon modes in high (red) and low (blue) temperature phases at the (Γ) point. (d, e) Temperature‐dependent X‐ray diffraction results which showcase the emergence of new Bragg's peaks below the structural transition.

If a THz wave (E_in_) is incident on a sample having a refractive index ‘n’ and thickness ‘d’ at temperature T, then the transmitted THz wave can be expressed as: $E_{\mathrm{out}}\left(\omega ,\;T\right)=E_{\mathrm{in}}\left(\omega ,\;T\right).\;e^{\frac{i\omega }{c}n\left(\omega ,\;T\right)d}$. The phase shift introduced by propagation through the sample relative to free space is then, $\phi \left(\omega ,\;T\right)=\frac{\omega d[n(\omega ,\;T)-1]}{c}.$ Since, the refractive index is related to dielectric function and if we consider the phase shift (ϕ_1_ and ϕ_2_) at two different temperature T_1_ and T_2_, then the change in phase shift becomes, $\Delta \phi =\phi_{2}-\phi_{1}=\frac{\omega d[\sqrt{\varepsilon (\omega ,\;T_{2})}-\sqrt{\varepsilon (\omega ,\;T_{1})}]}{c}$. This expression clearly shows that the phase shift directly depends on the dielectric function of the material. Further, the dielectric function in the THz regime can be expressed using a Lorentz oscillator model, $\tilde{\varepsilon }\left(\omega \right)=1+\omega_{p}^{2}\;\sum_{j}\frac{f_{j}}{\left(\omega_{oj}^{2}-\omega^{2}-i\omega \gamma \;\right)}$, where ω_p_, ω, ω_oj_, f_j_, and γ are the plasma frequency, driving field frequency, resonance frequency, oscillator strength, and damping coefficient, respectively. Clearly, the dielectric function can be influenced by the behavior of the phonon frequency. We performed phonon calculations for Fe_4_Nb_2_O_9_ in both low‐temperature (C2/c) and high‐temperature (P‐3c1) phases. The calculations were performed using the projector‐augmented wave method [44, 45], implemented in the Vienna Ab initio Simulation Package [46], with a plane wave energy cutoff of 450 eV. The exchange‐correlation interactions were treated using the generalized gradient approximation of Perdew–Burke–Ernzerhof [47], which was employed to fully relax the lattice structure. Atomic forces were kept below 1 meV/Å, and a 9 × 9 × 2 k‐point mesh was used to sample the Brillouin zone. To determine the phonon spectrum, a 2 × 2 × 1 supercell was constructed, and the frozen phonon method was applied using the PHONOPY package [48]. As shown in Figure 3a–c, the results indicate distinct phonon spectra for each crystalline phase, substantiating the impact of structural transitions on lattice vibrations. The transition in crystal structure leads to changes in force constants and symmetry, directly influencing the phonon dispersion and, thereby, the dielectric function of Fe_4_Nb_2_O_9_. It is noted that no phonon modes are observed in either temperature phase within our measured low‐frequency THz range (0.1–1 THz). However, within the Lorentz oscillator framework, we show that the emergence of new phonon modes and/or the renormalization of existing modes due to magnetic ordering can still significantly modify the dielectric response in this frequency range through their higher‐frequency tail (background contribution, Section SC6 C).

Experimentally, to determine the dielectric constant in the THz spectrum, the transmittance (*T*) is calculated (transmission of THz radiation with and without the sample),

(1) $$T=\frac{E\;\left(\omega ,\;T_{s}\right)}{E\;\left(\omega ,\;T_{r}\right)}=\frac{4\tilde{n}}{{\left(1+\tilde{n}\right)}^{2}}e^{\left(\frac{i\omega d\left(\tilde{n}-1\right)}{c}\right)}$$

where, E is the THz spectral amplitude, ω is the frequency, T_s_ and T_r_ are sample and reference temperatures, $\tilde{n}$ is the complex refractive index of the sample, d is the thickness of the sample, and c is the speed of light. Once n (real refractive index) and k (imaginary refractive index) are obtained, the dielectric function is obtained as follows: the real dielectric function is given by n^2^‐ k^2^, and the imaginary dielectric function is given by 2nk. In Figure 2c, we present the dielectric function at 0.2 THz during both the warming and cooling cycles, revealing transitions at approximately 93 K (both in warming and cooling), as well as at 77 K (warming) and 70 K (cooling, Inset Figure 2c). These transition temperatures correspond to the magnetic and structural phase transitions in Fe_4_Nb_2_O_9_ [40, 41, 42]. This behavior of the dielectric function closely traces the trend observed in the peak position plot (Figure 1c), indicating a strong link between the dielectric properties and the phase transitions. Furthermore, the dielectric function exhibits a dependence on the applied magnetic field (Figure 2d), indicating magnetodielectric coupling within the THz spectrum. This behavior can be understood in terms of spin–phonon coupling and has also been cited as a probable reason for kHz dielectric anomaly during magnetic and structural transitions [49]. The magnetic exchange interaction depends on the lattice displacement (u) and can be expanded as,

(2) $$J\;\left(u\right)=J_{0}+\frac{\partial J}{\partial u}u+\frac{1}{2}\frac{\partial^{2}J}{\partial u^{2}}u^{2}+\cdots ,$$

Using Equation (2) in the Heisenberg Hamiltonian gives,

(3) $$H_{\mathrm{spin}}=J\left(u\right)\;S_{i}.S_{j}=\left(J_{0}+\frac{\partial J}{\partial u}u+\frac{1}{2}\frac{\partial^{2}J}{\partial u^{2}}u^{2}+\cdots \right)\;S_{i}.S_{j},$$

Since phonon frequencies are determined by the second derivative of the potential energy, the quadratic term leads to a spin–phonon interaction term,

(4) $$\Rightarrow \;H_{\mathrm{spin}-\mathrm{phon}\mathrm{on}}=\frac{1}{2}\frac{\partial^{2}J}{\partial u^{2}}u^{2}S_{i}.S_{j},$$

This interaction modifies the effective lattice restoring force constant,

(5) $$\Rightarrow \;k_{\mathrm{effe}\mathrm{ctive}}=k+\frac{1}{2}\frac{\partial^{2}J}{\partial u^{2}}\langle S_{i}.S_{j}\rangle ,$$

Which leads to a renormalization of the phonon frequency,

(6) $$\Rightarrow \;\;\omega_{eff}=\sqrt{\omega_{o}^{2}+\frac{1}{2M}\frac{\partial^{2}J}{\partial u^{2}}<S_{i}.S_{j}>,}$$

**FIGURE 4:**
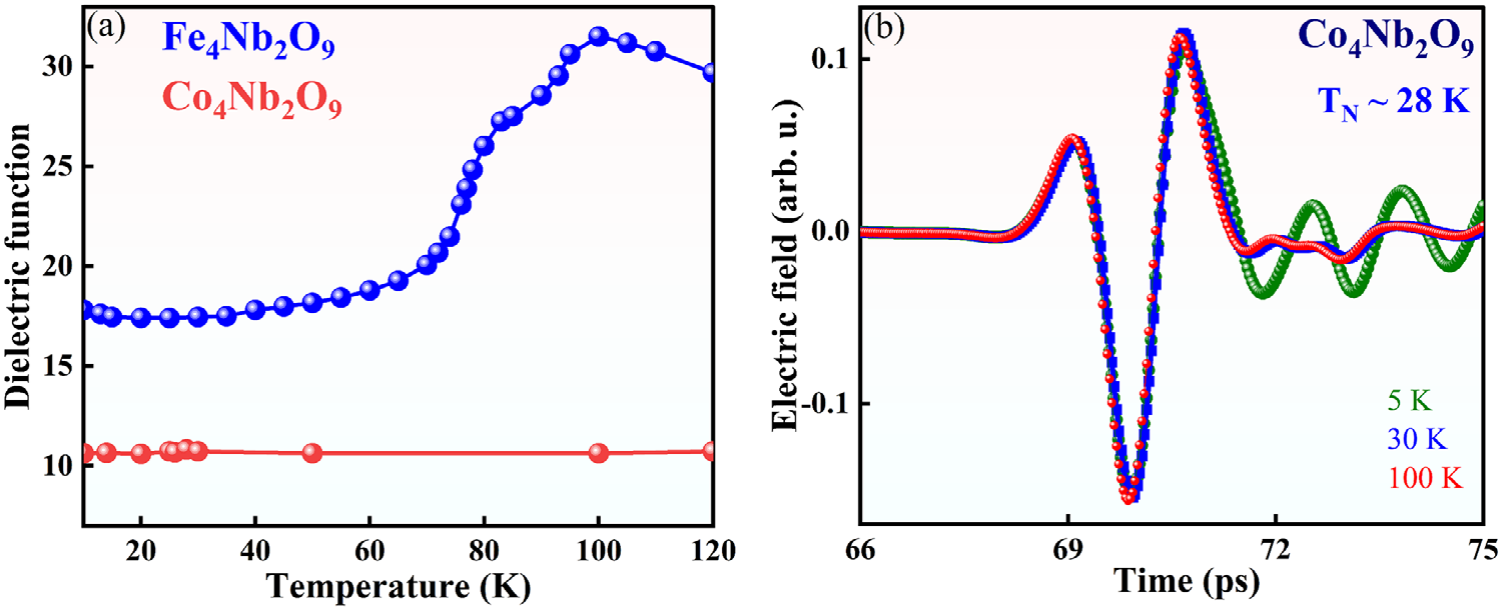
(a) Comparison of dielectric function vs. temperature of Fe_4_Nb_2_O_9_ and Co_4_Nb_2_O_9_ at 0.2 THz, (b) THz waveform of Co_4_Nb_2_O_9_ at different temperatures illustrating no THz time shift.

Thus, below 93 K, in Fe_4_Nb_2_O_9_, the onset of magnetic ordering modifies the spin correlation term (Equation 6), which consequently renormalizes the phonon frequencies and leads to a change in the dielectric response of the material ($\tilde{\varepsilon }\left(\omega \right)$). Upon further cooling, a structural transition occurs around 77 K (from trigonal P‐3c1  to monoclinic C2/c), as evidenced by temperature‐dependent X‐ray diffraction measurements (Figure 3d,e), in agreement with neutron diffraction results [41]. This structural transition alters the magnetic exchange interactions, thereby inducing additional changes in the phonon frequencies and further influencing the dielectric function (Figure 2a,b; Section SC6). The combined effect of these transitions results in a significant modulation of the dielectric function, which directly governs the observed THz phase shift (Δϕ). Furthermore, when an external field is applied to Fe_4_Nb_2_O_9_, it is the combination of spin‐phonon coupling and spin correlation function which modulates the dielectric function and thereby provides the magnetic field tuneability of phase shift (Figure 2b,d). This mechanistic link is further supported by comparing the behavior with Co_4_Nb_2_O_9_, a related magnetoelectric compound [50], which displays minimal dielectric variation with temperature (Figure 4a) and no notable THz waveform shifts (Figure 4b), underscoring the unique spin‐phonon coupling and dielectric sensitivity of Fe_4_Nb_2_O_9_.

**FIGURE 5:**
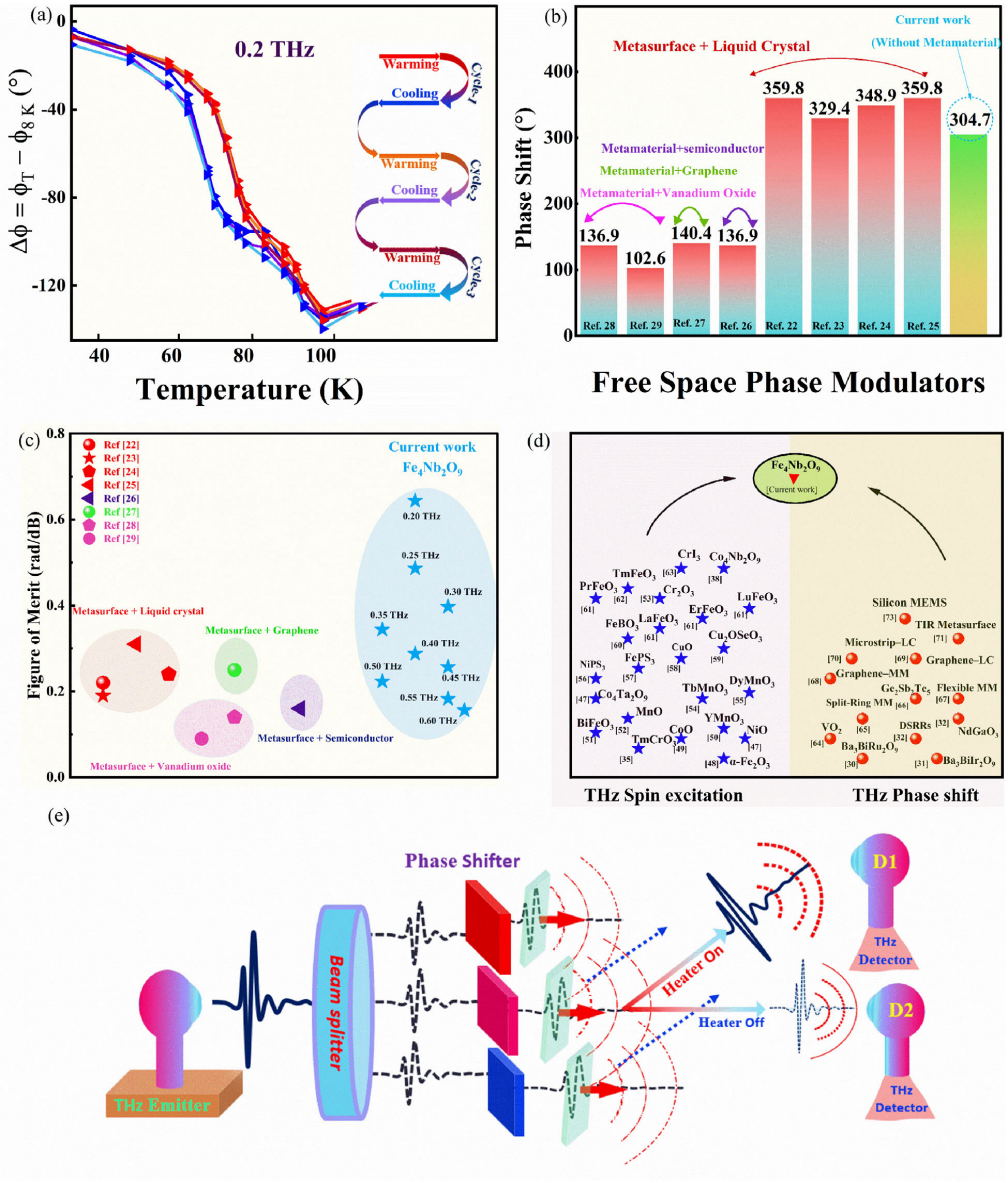
(a) Thermal repeatability of phase shift in Fe_4_Nb_2_O_9_ during three consecutive warming and cooling cycles at 0.2 THz. (b) Comparison of existing metasurface‐based free‐space modulators across various representative system classes reveals that the present work offers a phase modulator matching or exceeding π/2, operating in the sub‐THz region. (c) Comparison of the Figure of Merit for Fe_4_Nb_2_O_9_ with existing metasurface‐based free‐space modulators in the frequency range from 0.1 to 0.6 THz (Refs. [22, 23, 24, 25, 26, 27, 28, 29]). (d) Multifunctionality of Fe_4_Nb_2_O_9_ in the THz frequency range compared to other existing materials for THz spin excitation and phase shift. (e) Proof‐of‐concept of THz phased array antenna based on Fe_4_Nb_2_O_9_.

To examine the thermal stability and practical applicability of Fe_4_Nb_2_O_9_, temperature‐dependent THz measurements were performed over three successive heating and cooling cycles. As shown in Figure 5a, the phase shift completely overlaps in all three cycles, indicating excellent reproducibility. In addition, the substantial phase shift demonstrated in this work using only an intrinsic material (without a metasurface) exceeds or is comparable to the phase shifts achieved with artificially structured metasurfaces in the sub‐THz band with a high Figure of Merit (Figure 5b,c). Thus, the current work signifies a paradigm shift, paving the way for intrinsic material‐based THz devices, that too without the need for any expensive cleanroom fabrications. As wireless systems become increasingly widespread, the demand for higher data rates and broader bandwidths drives a shift toward higher carrier frequencies, particularly in the THz range [1, 2, 3, 4, 5, 6, 51, 52, 53]. An important advantage at these frequencies is the reduced angular divergence of THz radiation due to diminished diffraction effects at shorter wavelengths‐leading to enhanced directionality. Consequently, mechanical rotation of the radiation source becomes impractical for beam steering; instead, beam‐steering strategies are essential to control the propagation direction of THz waves (Figure 5e). Leveraging our intrinsic THz phase shifter, Fe_4_Nb_2_O_9_, with a maximum tunability of 304.7°, we propose a proof‐of‐concept proposition of *“THz phase array antenna.”* This system comprises Fe_4_Nb_2_O_9_‐based phase shifters arranged in parallel configurations and maintained at different temperatures to introduce precise phase differences. When all elements are at the same temperature, no relative phase shift occurs, aligning the wave propagation with the incident direction. However, by varying temperatures across the array, relative phase shifts are induced among the elements. This alters the wavefront direction, steering the THz beam at a desired angle, as the propagation direction is perpendicular to the wavefront. This approach offers a dynamic, non‐mechanical method for beam steering in future THz communication systems. Further, it has potential for implementing THz flat optics without metasurfaces, hence easing out fabrication efforts tremendously.

**FIGURE 6:**
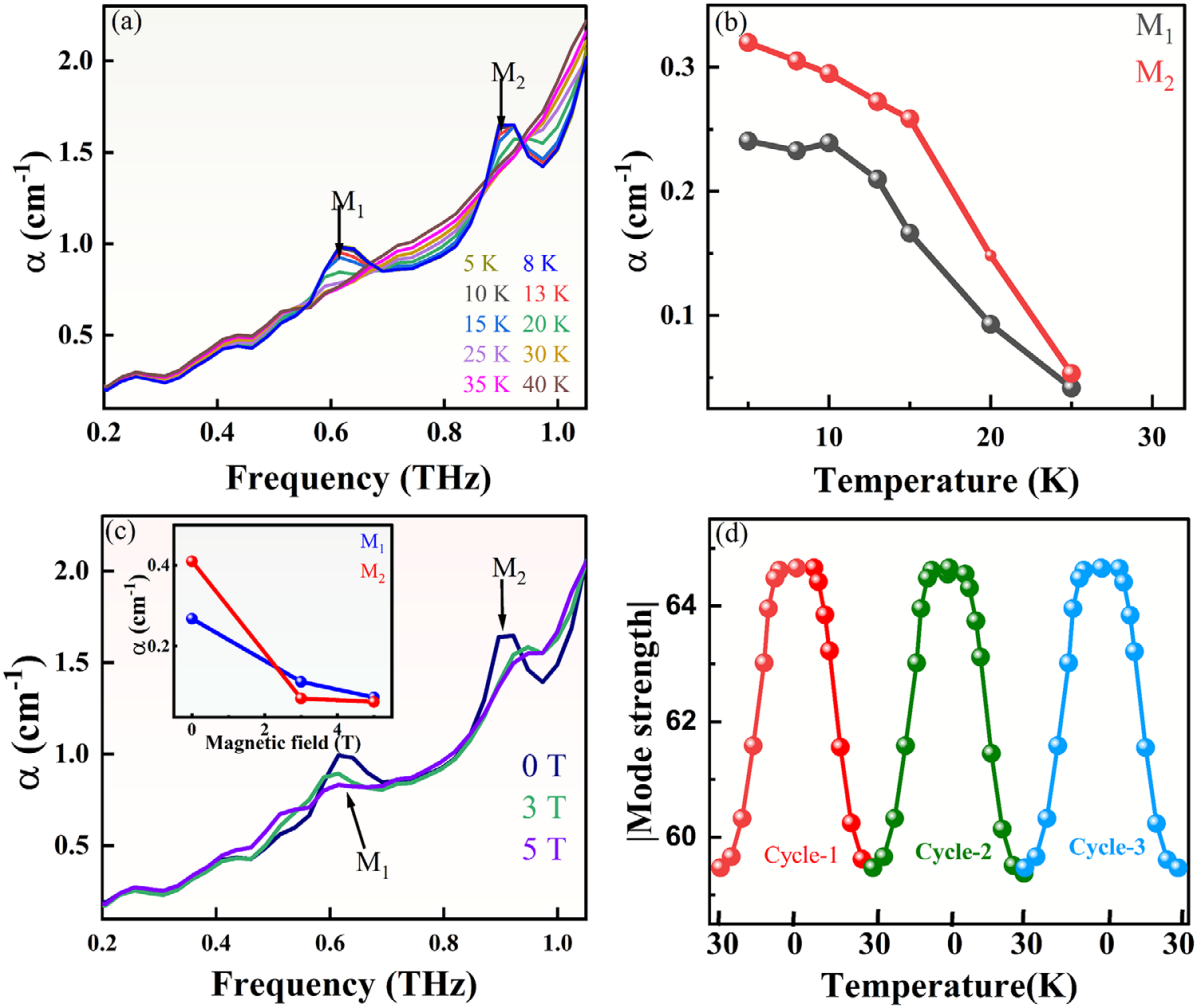
(a) Absorption coefficient (α) as a function of THz frequency with varying temperature. (b) Variation in the peak strength of mode M_1_ and M_2_ with temperature. (c) Variation in α with magnetic field (Inset shows the variation in peak intensity of mode M_1_ and M_2_ with magnetic field). (d) Testing repeatability of spin‐excitation M_1_ in three consecutive warming and cooling cycles.

In addition to the temperature and magnetic field‐induced phase shifts observed within the 6G relevant sub‐THz band, we identify two distinct excitations, labeled M_1_ and M_2_, at 0.62 and 0.91 THz in the THz spectrum. These excitations emerge below the magnetic transition temperature, and their strength increases as the temperature decreases, strongly indicating their magnetic origin (Figure 6a,b). Upon application of an external magnetic field, the intensities of these excitations diminish with increasing field strength, with the M_1_ mode shifting towards lower THz frequencies, as shown in Figure 6c. In Fe_4_Nb_2_O_9_, the magnetic Fe ions occupy two inequivalent crystallographic sites (Fe1 and Fe2) located at the *f* Wyckoff position with multiplicity 8 in the low temperature monoclinic (C2/c) structure [42]. Thus, the crystallographic unit cell contains 16 Fe ions. However, due to crystal symmetry, these correspond to 8 independent magnetic ions. Since the magnetic propagation vector is k = (0,0,0), the magnetic and crystallographic unit cells are identical. According to the Linear Spin Wave theory, the number of magnon branches equals the number of independent magnetic ions in the unit cell. Therefore, 8 magnons are expected at the Brillouin zone center. One of these modes corresponds to a low‐energy gapless/Goldstone mode associated with the spontaneous breaking of spin rotational symmetry below the magnetic ordering temperature, which has been observed in this class of family [36, 38, 54]. While the remaining modes are gapped magnons whose energies are determined by the exchange interactions and magnetic anisotropy in the system [36, 38]. Out of these, two of these gapped magnons modes (M_1_ and M_2_) are visible in our THz spectrum. We also carried out preliminary spin‐wave calculations using the SpinW package [55], which indicate the presence of both a gapless and gapped magnon modes in this material (Section SC7). However, a comprehensive determination of the magnon spectrum in the whole momentum space will require future investigations using Inelastic Neutron Scattering. For practical relevance, we tested the repeatability of the magnon mode, demonstrating consistent behavior, as shown in Figure 6d. The presence of magnons in the THz frequency range is highly desirable because they are fundamental to magnon‐based computation, which avoids Joule heating, unlike traditional electronic circuits. Observing magnons at these frequencies in Fe_4_Nb_2_O_9_ holds promise for exploring phenomena such as magnon sum and difference processes in the THz spectrum‐a rapidly emerging field known as THz magnon algebra [11, 36, 56, 57]. Recent studies, such as those in YFeO_3_ using advanced two‐dimensional THz spectroscopy, highlight the potential for THz‐based manipulation and detection of spin dynamics in magnetic materials, with Fe_4_Nb_2_O_9_ offering exciting new possibilities in this domain. THz magnetism and spintronics encompass multiple facets in their rapidly evolving research areas with broad technological applications. Materials having THz‐active multifunctional properties are highly sought after for advancing next‐generation devices. In Figure 5d, we present a comparative analysis of our findings with existing literature [30, 31, 32, 36, 58, 59, 60, 61, 62, 63, 64, 65, 66, 67, 68, 69, 70, 71, 72, 73, 74, 75, 76, 77, 78, 79, 80, 81, 82, 83, 84, 85], highlighting Fe_4_Nb_2_O_9_ as a standout multifunctional model material. It uniquely demonstrates the notion of coexistence of two critical THz properties‐intrinsic phase shifting and spin excitations. This work serves as an inspiration for future efforts to identify and develop multifunctional THz materials critical for integrated technological innovations.

## 3 Conclusions

In conclusion, we experimentally demonstrate, for the first time, the coexistence of intrinsic THz phase shifting and spin excitations within the same material system. Fe_4_Nb_2_O_9_ emerges as a model THz multifunctional active material, showcasing a large intrinsic phase shift of 304.7° at 0.56 THz (30–93 K) across a broad bandwidth (0.1–0.56 THz), with rare magnetic field tunability. Corroborated by theoretical lattice vibrational calculations, the observed phase shift is attributed to dielectric modulation driven by the phase transitions within the system. The huge intrinsic phase shift has the potential of revolutionizing THz flat optics by implementing a beam steerer, a flat lens without involving complex metasurfaces. In addition, at 8 K, two magnon excitations at 0.62 and 0.91 THz are identified, highlighting its relevance to THz magnonics. This unprecedented synergy positions Fe_4_Nb_2_O_9_ as a role‐model material, inspiring the discovery of further multifunctional THz quantum materials to unify communication and computation technologies.

## Conflicts of Interest

The authors declare no conflicts of interest.

## Supporting information




**Supporting File**: advs75555‐sup‐0001‐SuppMat.docx.

## Data Availability

The data that support the findings of this study are available from the corresponding author upon reasonable request.
