# Supplementary material for: Concurrent Terahertz Spin Excitations and Phase Shift Control in Fe4Nb2O9: A Material for Synergizing Computation and Communication Technologies
Source: Adv Sci (Weinh). 2026 May 13;13(42):e75555. doi: 10.1002/advs.75555 (PMC13336138; doi:10.1002/advs.75555)
Supplement: Supplementary file 1 — Supporting File: advs75555‐sup‐0001‐SuppMat.docx. [file ADVS-13-e75555-s001.docx]

**Supplementary Information**

**Concurrent Terahertz Spin Excitations and Phase Shift Control in Fe_4_Nb_2_O_9_: A Material for Synergizing Computation and Communication Technologies**

Brijesh Singh Mehra^1#^, Karan Datt Sharma^1#^, Sanjeev Kumar^1^, Gaurav Dubey^1^, Mitanshi Gupta^1^, Ravi Shankar Singh^1^, Dibakar Roy Chowdhury^4^, Christine Martin^2^, Antoine Maignan^2^, Kiran Singh^3^, Dhanvir Singh Rana^1,*^

***^1^****Department of Physics, Indian Institute of Science Education and Research Bhopal, Bhopal, Madhya Pradesh 462066, India*

***^2^****CRISMAT, Laboratoire de Cristallographie et Sciences des Matériaux, UMR6508, Normandie Université, ENSICAEN, UNICAEN, CNRS, 14050 Caen, France*

***^3^****Department of Physics, Dr. B. R. Ambedkar National Institute of Technology, Jalandhar, Punjab 144011, India*

*^4^School of Engineering, Anurag University, Ghatkesar, Telangana 500088, India*

**dsrana@iiserb.ac.in, #equal contribution*

**C1: Sample Detail:**

A polycrystalline sample (~2 × 2 × 0.7 mm^3^) of Fe_4_Nb_2_O_9_ was synthesized via a solid-state reaction method and was taken from reference 40.

**C2: Terahertz Spectroscopy:**

THz time-domain spectroscopy measurements were performed using a Tera K15 Menlo system, integrated with an Oxford SpectromagPT cryostat in Faraday geometry [Figure S 1]. The THz beam path was continuously purged with dry nitrogen to reduce absorption caused by atmospheric humidity. To enhance the signal-to-noise ratio, 3000 THz waveforms were averaged at each temperature and magnetic field condition. The collected time-domain signals were Fourier transformed into the frequency domain using Fast Fourier Transform, allowing analysis of both phase and amplitude of the THz waves. Additionally, optical parameters such as the refractive index and dielectric constant within the THz range were extracted by comparing transmission spectra recorded with and without the sample. Absorption Coefficient was calculated by, $\alpha\left( \omega, T \right)=-\frac{2}{d}\log_{e} \frac{E_{sample}(\omega, T)}{E_{reference}(\omega, T)}$, where d is the thickness of the sample, E_sample_ and E_reference_ are the spectral amplitude with and without the sample, respectively.


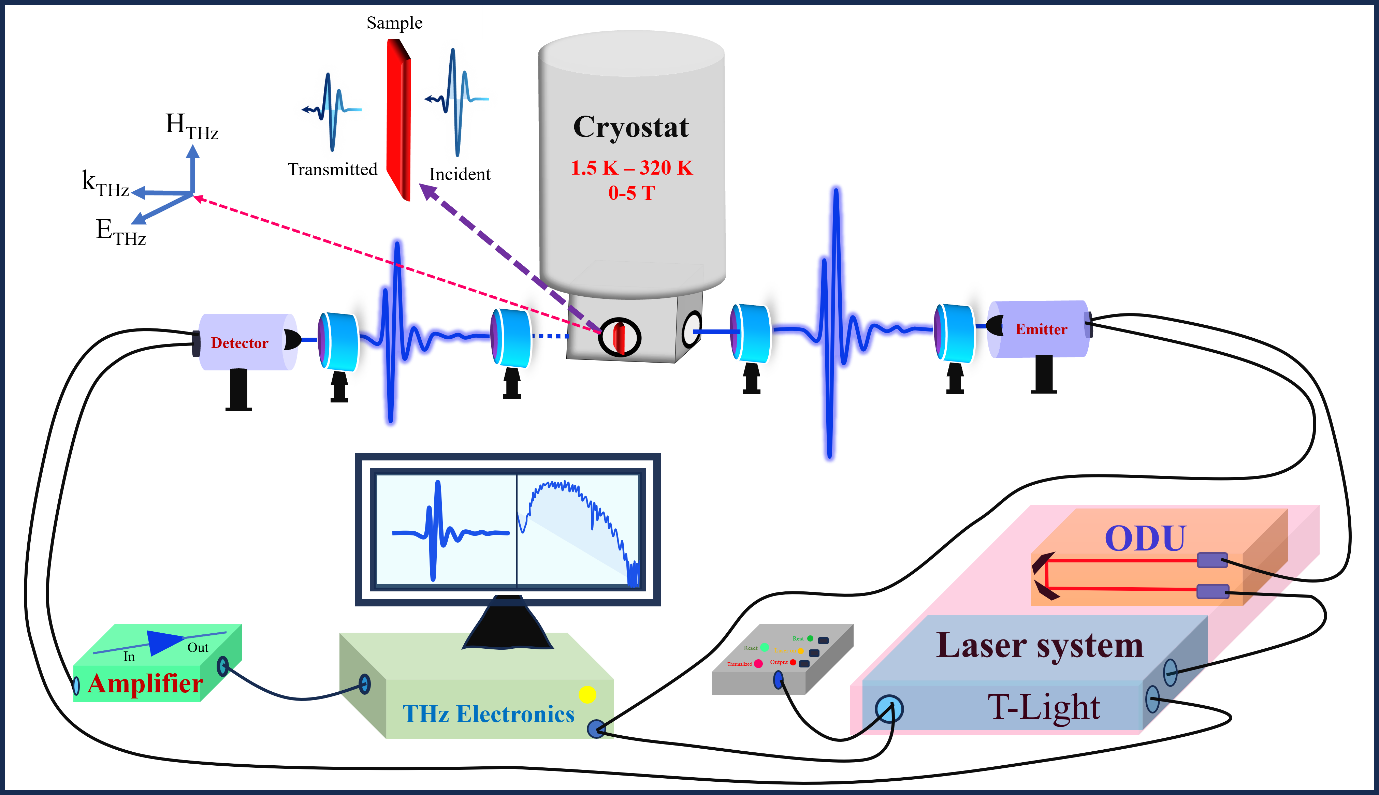


***Figure S 1:*** *Magneto-THz spectroscopy experimental setup.*

**
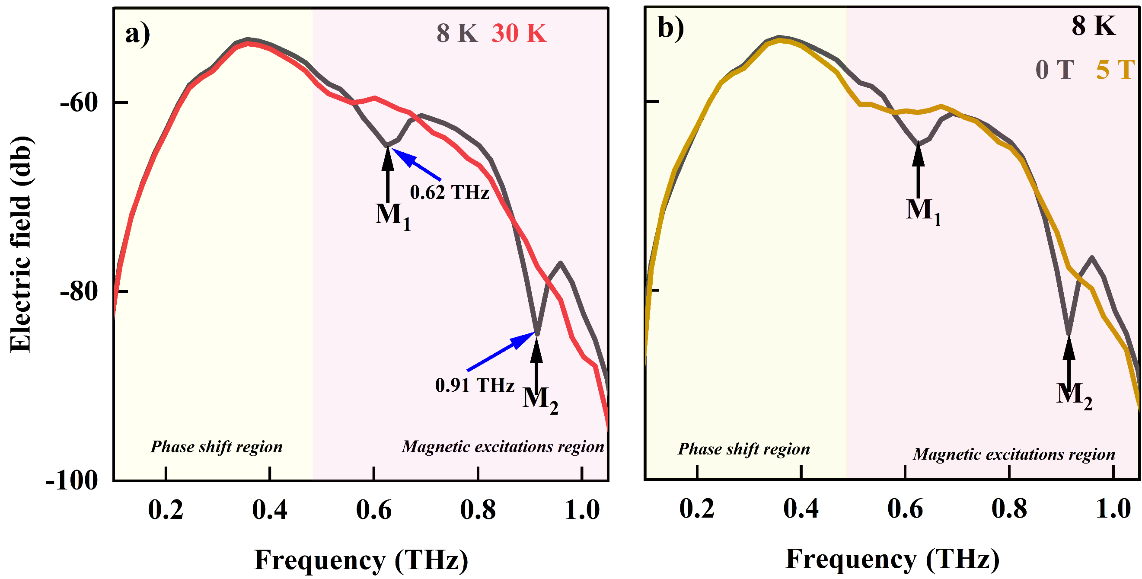
**

***Figure S 2:*** *a) Temperature dependent, b) magnetic field dependent terahertz electric field (FFT) at 8 K. The frequency range is divided into two regions: i) phase shifting (yellow) and ii) magnon excitation (red).*


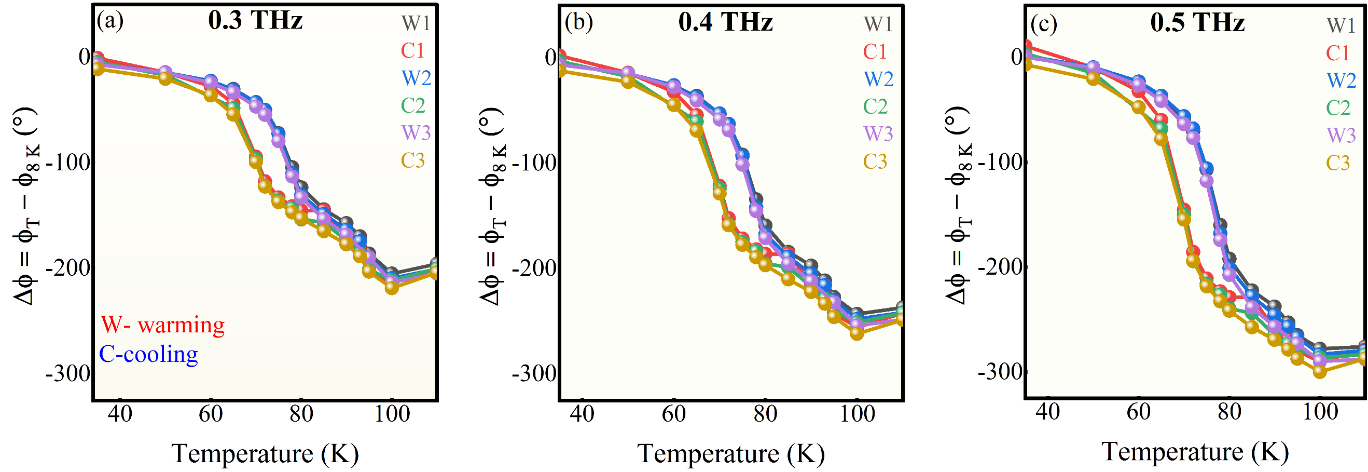


***Figure S 3:*** *Thermal repeatability of the compound in three consecutive warming and cooling cycles: a) 0.3 THz, b) 0.4 THz, c) 0.5 THz.*





***Figure S 4:*** *(a)The magnetic-field-dependent terahertz electric field (FFT) at 20 K remains unchanged with varying magnetic field, (b) comparison of phase shift with magnetic field at two different temperatures (8 K and 20 K).*

**C3: Gamma Point Phonon Energies calculation**

|  | High Temp. Phase | Low Temp. Phase |  |
| --- | --- | --- | --- |
|  | Band Gap = 3.28 eV | Band Gap = 3.31 eV |  |
| Mode No. | Energy (THz) | Energy (THz) | Mode No. |
| 1 | 1.940120 | 1.917065 | 1 |
|  |  | 1.931165 | 2 |
| 2 | 3.160526 | 3.141362 | 3 |
|  |  | 3.149570 | 4 |
| 3 | 3.657994 | 3.628933 | 5 |
|  |  | 3.642422 | 6 |
| 4 | 4.262457 | 4.215043 | 7 |
| 5 | 5.038576 | 4.879110 | 8 |
| 6 | 5.772315 | 5.664498 | 9 |
| 7 | 6.103601 | 6.029258 | 10 |
|  |  | 6.039415 | 11 |
| 8 | 6.462327 | 6.331502 | 12 |
| 9 | 6.464612 | 6.337886 | 13 |
| 10 | 6.581096 | 6.544083 | 14 |
|  |  | 6.549037 | 15 |
| 11 | 6.640542 | 6.636182 | 16 |
|  |  | 6.636784 | 17 |
| 12 | 7.002210 | 6.948736 | 18 |
| 13 | 7.067026 | 6.972472 | 19 |
| 14 | 7.087807 | 7.090134 | 20 |
|  |  | 7.099866 | 21 |
| 15 | 7.342924 | 7.301932 | 22 |
| 16 | 7.638605 | 7.545407 | 23 |
|  |  | 7.546706 | 24 |

***Table 1****: Phonon calculations in high and low temperature phases using frozen-phonon method within nonmagnetic DFT+U (for U_Fe_ = 8eV, k-mesh:9×9×2, supercell- 2×2×1).*

**C4: Comparison with existing functional material in the literature**

| S.N. | Materials | Spin excitations | Phase shift | References |
| --- | --- | --- | --- | --- |
| 1 | **Fe_4_Nb_2_O_9_** | ✔ | ✔ | Current work |
| 2 | NiO | ✔ | ✗ | 47 |
| 3 | α-Fe_2_O_3_ | ✔ | ✗ | 48 |
| 4 | FeBO_3_ | ✔ | ✗ | 60 |
| 5 | CoO | ✔ | ✗ | 49 |
| 6 | YMnO_3_ | ✔ | ✗ | 50 |
| 7 | BiFeO_3_ | ✔ | ✗ | 51 |
| 8 | MnO | ✔ | ✗ | 52 |
| 9 | TbMnO_3_ | ✔ | ✗ | 54 |
| 10 | Cr_2_O_3_ | ✔ | ✗ | 53 |
| 11 | DyMnO_3_ | ✔ | ✗ | 55 |
| 12 | NiPS_3_ | ✔ | ✗ | 56 |
| 13 | FePS_3_ | ✔ | ✗ | 57 |
| 14 | CuO | ✔ | ✗ | 58 |
| 15 | Cu_2_OSeO_3_ | ✔ | ✗ | 59 |
| 16 | TmCrO_3_ | ✔ | ✗ | 35 |
| 17 | LaFeO_3_ | ✔ | ✗ | 61 |
| 18 | PrFeO_3_ | ✔ | ✗ | 61 |
| 19 | ErFeO_3_ | ✔ | ✗ | 61 |
| 20 | LuFeO_3_ | ✔ | ✗ | 61 |
| 21 | TmFeO_3_ | ✔ | ✗ | 62 |
| 22 | Co_4_Ta_2_O_9_ | ✔ | ✗ | 46 |
| 23 | Co_4_Nb_2_O_9_ | ✔ | ✗ | 38 |
| 24 | CrI_3_ | ✔ | ✗ | 63 |
| 25 | Ba_3_BiRu_2_O_9_ | ✗ | ✔ | 30 |
| 26 | Ba_3_BiIr_2_O_9_ | ✗ | ✔ | 31 |
| 27 | VO_2_ | ✗ | ✔ | 64 |
| 28 | Split-Ring MM | ✗ | ✔ | 65 |
| 29 | GeSbTe/ Ge_2_Sb_2_Te_5_ (GST) | ✗ | ✔ | 66 |
| 30 | NdGaO_3_ | ✗ | ✔ | 32 |
| 31 | Flexible MM Phase Shifter | ✗ | ✔ | 67 |
| 32 | Graphene–Metal Metasurface Shifter | ✗ | ✔ | 68 |
| 33 | Graphene–LC Phase Shifter | ✗ | ✔ | 69 |
| 34 | Microstrip–LC Phase Shifter | ✗ | ✔ | 70 |
| 35 | Tunable TIR Metasurface Shifter | ✗ | ✔ | 71 |
| 36 | DSRRs | ✗ | ✔ | 72 |
| 37 | Silicon MEMS | ✗ | ✔ | 73 |

***Table 2****: Multifunctionality of Fe_4_Nb_2_O_9_ in the THz frequency range compared to other existing materials for THz spin excitation and phase shift.*

**C5: Figure-of-Merit Table**

| **S.N.** | **Frequency (THz)** | **FoM (rad/dB)** |
| --- | --- | --- |
| 1 | 0.111 | 0.85535 |
| 2 | 0.133 | 1.13332 |
| 3 | 0.156 | 1.21569 |
| 4 | 0.178 | 0.73843 |
| 5 | 0.200 | 0.64386 |
| 6 | 0.222 | 0.57019 |
| 7 | 0.245 | 0.486433 |
| 8 | 0.267 | 0.451884 |
| 9 | 0.289 | 0.427125 |
| 10 | 0.312 | 0.39686 |
| 11 | 0.334 | 0.36612 |
| 12 | 0.356 | 0.34372 |
| 13 | 0.378 | 0.31522 |
| 14 | 0.401 | 0.28732 |
| 15 | 0.423 | 0.27278 |
| 16 | 0.445 | 0.25666 |
| 17 | 0.468 | 0.23549 |
| 18 | 0.490 | 0.22304 |
| 19 | 0.512 | 0.21815 |
| 20 | 0.534 | 0.19692 |
| 21 | 0.557 | 0.1825 |

***Table 3****: Figure of merit (FoM) for different frequencies in the terahertz band from 0.111 THz to 0.579 THz.*





***Figure S 5:*** *Loss with respect to 30 K with a) temperature for different frequencies, b) frequency at different temperatures.*

As observed from the measurements, the loss is higher above the magnetic transition temperature, which is also consistent with the THz time-domain waveforms showing stronger attenuation in this temperature regime compared to the magnetically ordered phase [Fig.1 (a, b) main manuscript]. This enhanced loss can be attributed primarily to increased dielectric absorption arising from phonon damping and spin-disorder-induced scattering in the paramagnetic state.

Minimizing loss in Fe_4_Nb_2_O_9,_ therefore, requires careful dielectric optimization. One effective strategy is compositional tuning within the A_4_B_2_O_9_ family (A = Fe, Co, Ni; B = Nb, Ta), where substitution at the A- and/or B-sites can systematically modify the lattice dynamics, magnetic exchange interactions, and phonon damping. Such tuning enables optimization of the dielectric response by reducing the imaginary part of the dielectric function (absorption) while maintaining sufficient real-part modulation for phase shifting.

**C6: Lorentz dielectric model**

**A) Relation of Phase Shift and Dielectric Function:**

When a THz wave is incident (E*_in_*) on a sample having a dielectric $\tilde{}$ and thickness ‘d’ at temperature T, the transmitted THz wave can be expressed as:

$E_{out}\left( , T \right)= E_{in}\left( , T \right). e^{\frac{i}{c}\sqrt{\tilde{}\left( , T \right)}d}$, (1)

The phase shift introduced by propagation through the sample relative to free space is then,

$\left( , T \right)=\frac{d\left[ \sqrt{\tilde{}\left( , T \right)}-1 \right]}{c}$, (2)

If the phase shift (φ_1_ and φ_2_) is evaluated at two different temperature T_1_ and T_2_, then the corresponding change in phase shift becomes,

$={}_{2}-{}_{1}=\frac{d\left[ \sqrt{\tilde{}\left( , T_{2} \right)}-\sqrt{\tilde{}\left( , T_{1} \right)} \right]}{c}$, (3)

This expression clearly shows that the phase shift directly depends on the dielectric function of the material. Further, the dielectric function in the THz regime can be expressed using a simple Lorentz oscillator model,

$\tilde{}\left( \right)=1+{}_{p}^{2} \sum_{j} \frac{f_{j}}{({}_{oj}^{2}-{}^{2}-i )}$, (4)

where ω_p_, ω, ω_oj_, f_j_, and γ are the plasma frequency, driving field frequency, resonance frequency, oscillator strength and damping coefficient respectively. Clearly, the dielectric function can be influenced by the behaviour of the phonon frequency.

**B) Theoretical Phonon Calculations *(No Phonon Modes in our observable THz window [0.1 to 1 THz])***

We performed phonon calculations for Fe_4_Nb_2_O_9_ in both low-temperature (C2/c) and high-temperature (P-3c1) phases. As shown in Figure 3(a-c) main manuscript, the results indicate distinct phonon spectra for each crystalline phase, substantiating the impact of structural transitions on lattice vibrations *(The number of phonon modes in lower-temperature region is much more than the high-temperature region)*. The transition in crystal structure leads to changes in force constants and symmetry, directly influencing the phonon dispersion and, thereby, the dielectric function of Fe_4_Nb_2_O_9_. It is noted that no phonon modes are observed in either temperature phase within our measured low-frequency THz range (0.1–1 THz).

**C) The effect of high frequency phonon modes in our observable low-frequency THz window*: Dielectric function using simple Lorentz Oscillator Model and experimental THz Dielectric function***

However, within the simple Lorentz oscillator framework, as shown in Figure S6 and S7, we show that the emergence of new phonon modes and/or the renormalization of existing modes due to magnetic ordering can still significantly modify the dielectric response in this frequency range through their higher-frequency tail (background contribution).


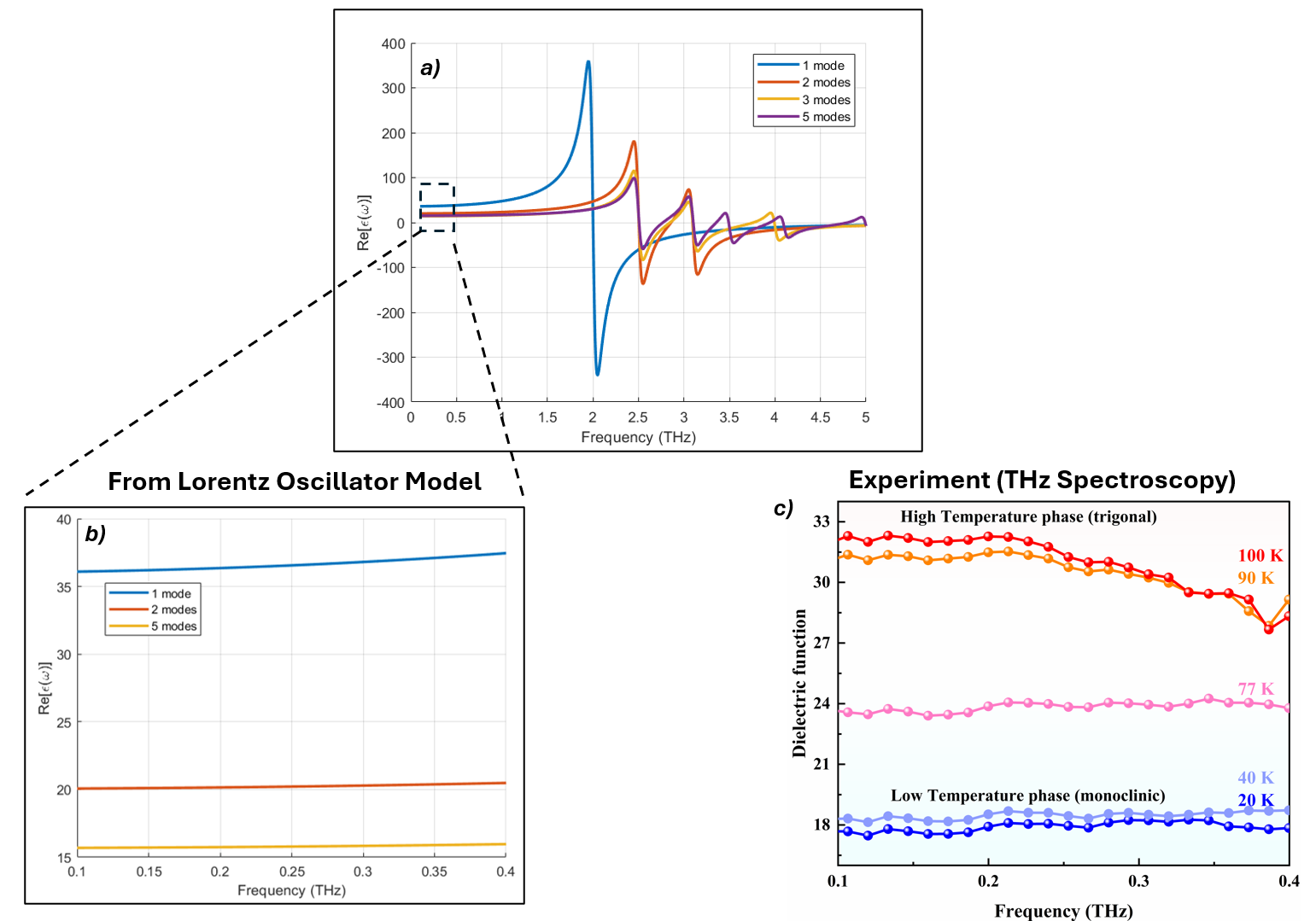


*Figure S 6: (a, b) Calculated dielectric function using Lorentz oscillator model in MATLAB. It shows how dielectric modulates with phonon frequency in the higher frequency region and its effect on the background in the low-frequency region as the number of phonon modes increases. c) Experimental THz dielectric function as the number of phonons increases as we go from high to low temperature phase.*

*
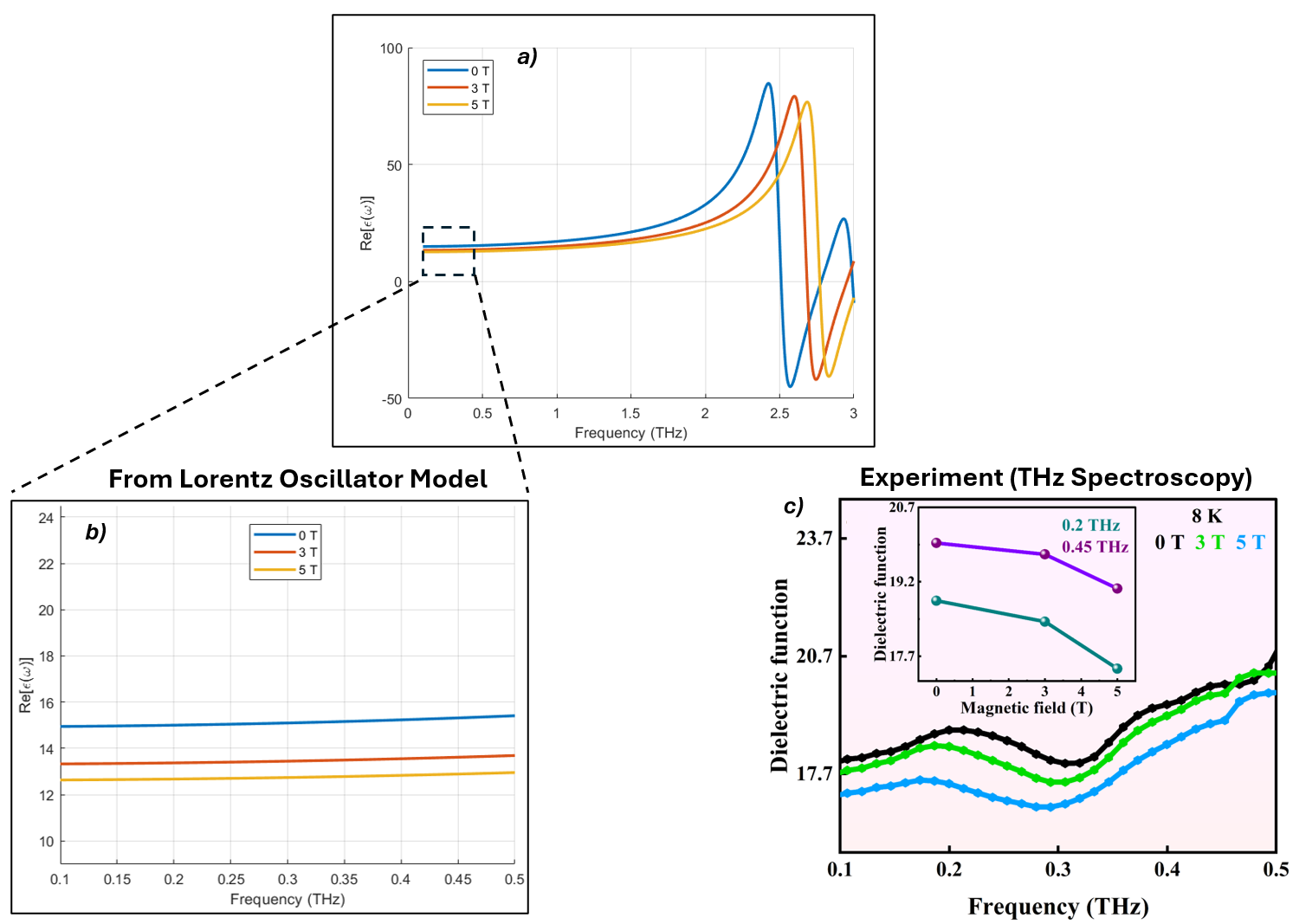
*

*Figure S 7: (a, b): Effect on the dielectric when the external magnetic field influences the phonon frequency in the high and low frequency THz region respectively. Here, equation (9) is approximated to ω(H) =ω_0_+λ’M(H)^2^, for calculation feasibility, where a simple magnetization model M(H)=tanh(H/H_0_) is taken. c) Experimental dielectric function in the low frequency THz region with increasing magnetic field.*

**D) Quantification of Phonon mode with Phase Transitions:**

Experimentally, the THz dielectric response of Fe_4_Nb_2_O_9_ exhibits a pronounced change across the magnetic (93 K) and structural phase transitions. This behaviour can be understood in terms of spin–phonon coupling and has also been cited as a probable reason for kHz dielectric anomaly during magnetic and structural transitions [1]. The magnetic exchange interaction depends on the lattice displacement (u) and can be expanded as,

$J \left( u \right)=J_{0}+ \frac{\partial J}{\partial u}u + \frac{1}{2}\frac{\partial^{2}J}{{\partial u}^{2}}u^{2}+\ldots$, (5)

Using equation (5) in the Heisenberg Hamiltonian gives,

H_spin_ =J(u) S_i_.S_j_ = ${(J}_{0}+ \frac{\partial J}{\partial u}u + \frac{1}{2}\frac{\partial^{2}J}{{\partial u}^{2}}u^{2}+\ldots$) $S_{i}S_{j}$, (6)

Since phonon frequencies are determined by the second derivative of the potential energy, the quadratic term leads to a spin–phonon interaction term,

⇒ H_spin-phonon_ = $\frac{1}{2}\frac{\partial^{2}J}{{\partial u}^{2}}u^{2}S_{i}S_{j}$, (7)

This interaction modifies the effective lattice restoring force constant,

⇒ k_effective_ = k + $\frac{1}{2}\frac{\partial^{2}J}{{\partial u}^{2}}<S_{i}S_{j}>$, (8)

which leads to a renormalization of the phonon frequency,

⇒ ${}_{eff}=\sqrt{{}_{o}^{2}+ \frac{1}{2M}\frac{\partial^{2}J}{{\partial u}^{2}}{<S}_{i}S_{j}>}$, (9)

**E) Full Picture:**

The theoretical framework presented above can be understood in the context of Fe_4_Nb_2_O_9_ as: Below 93 K, in Fe_4_Nb_2_O_9_, the onset of magnetic ordering modifies the spin correlation term (Eq. 9), which consequently renormalizes the phonon frequencies and leads to a change in the dielectric response of the material (Eq. 4). Upon further cooling, a structural transition occurs around 77 K, as evidenced by temperature-dependent X-ray diffraction measurements (Figure 3 (d, e) main manuscript). This structural transition alters the positions of the magnetic ions and modifies the magnetic exchange interactions, thereby inducing additional changes in the phonon frequencies and further influencing the dielectric function. The combined effect of these transitions results in a significant modulation of the dielectric function, which directly governs the observed THz phase shift (equation 3). Furthermore, it is the combination of spin-phonon coupling and spin correlation function which modulates the dielectric function when an external field is applied to Fe_4_Nb_2_O_9_ (equation 3), thereby giving us the magnetic field induced THz phase shift.

C7: **Spin-wave calculations:**

The spin-wave calculations were performed using the SpinW code developed by Sándor Tóth and B. Lake [6]. The lattice parameters used in the simulation were a = 9.0916, b=5.1826, c = 14.1983 and α= 90º, β = 91.677º, γ = 90º at 10 K, taken from Ref. 1. The crystal structure contains two inequivalent Fe sites located at coordinates (0.1645, 0.5011, 0.0132) and (0.1722, 0.5054, 0.3066). By applying the crystallographic symmetry operations, these two sites generate a total of 16 Fe magnetic ions in the unit cell with fractional coordinates (x, y, z).

'Fe1' 0.165 0.501 0.013

'Fe1' 0.665 0.001 0.013

'Fe1' 0.836 0.501 0.487

'Fe1' 0.336 0.001 0.487

'Fe1' 0.836 0.499 0.987

'Fe1' 0.336 0.999 0.987

'Fe1' 0.165 0.499 0.513

'Fe1' 0.665 0.999 0.513

'Fe2' 0.172 0.505 0.307

'Fe2' 0.672 0.005 0.307

'Fe2' 0.828 0.505 0.193

'Fe2' 0.328 0.005 0.193

'Fe2' 0.828 0.495 0.693

'Fe2' 0.328 0.995 0.693

'Fe2' 0.172 0.495 0.807

'Fe2' 0.672 0.995 0.807


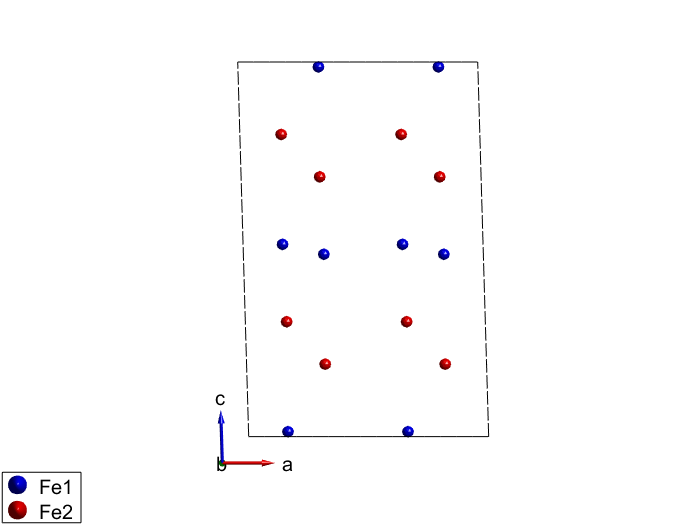


*Figure S 8: Generated unit cell of Fe_4_Nb_2_O_9_.*

The magnetic unit cell contains 16 Fe ions, and a total of eight magnetic exchange interactions (J1, J2, J3,…,J8) were defined using the SpinW code. Since, the in-plane anisotropy lies within the basal plane; therefore, the anisotropy parameter $D$ was taken to be larger than the exchange interactions associated with the out-of-plane directions, similar to what has been reported for the related compounds [5-7]. For simplicity, the in-plane anisotropy parameter $D$ was assumed to be the same for both inequivalent Fe sites (Fe1 and Fe2).

In total, nine parameters (eight exchange interactions and one anisotropy parameter) were involved in the model, whereas only two experimental data points corresponding to the observed magnon modes (M_1_ and M_2_) were available from the THz measurements. Therefore, the parameter set is not uniquely constrained, and the present calculation should be considered only as a preliminary attempt. More definitive determination of the magnetic interactions will require future Inelastic Neutron Scattering experiments. The calculated magnon energy dispersion along the high-symmetry direction ( Γ → M → K) exhibits several magnon branches at the Brillouin-zone center [Figure S 9 b]. The calculations indicate the presence of a low-energy Goldstone-like mode, consistent with the symmetry considerations discussed above, along with multiple gapped magnon excitations.

Therefore, based on the symmetry analysis and the preliminary spin-wave calculations, the experimentally observed modes M_1_ and M_2_ can be assigned to gapped magnon modes, while a Goldstone-like mode exists in Fe_4_Nb_2_O_9_ but is out of our instrument range.


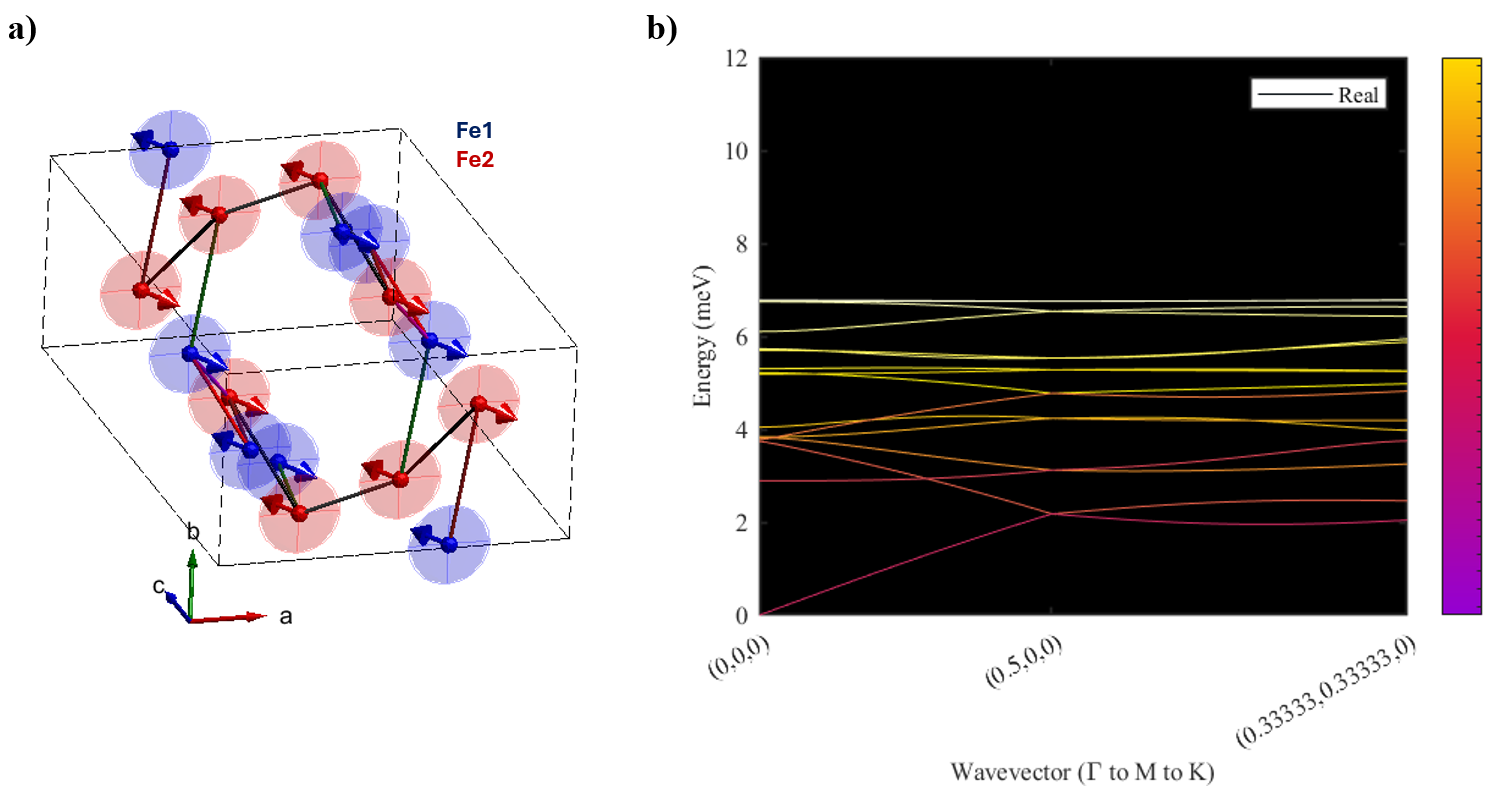


*Figure S 9: (a, b) Magnetic structure and calculated energy dispersion plot of Fe_4_Nb_2_O_9_. Caution: The energy dispersion plot is a preliminary attempt only. Future Inelastic Neutron Scattering experiments are required.*

**References:**

[1] R. Jana, D. Sheptyakov, X. Ma, J.A. Alonso, M. Pi, A. Muñoz, Z. Liu, L. Zhao, N. Su, S. Jin, X. Ma, K. Sun, D. Chen, S. Dong, Y. Chai, S. Li, and J. Cheng, Phys Rev B 100(9), 094109 (2019).

[2] A. Maignan, and C. Martin, Phys Rev B 97(16), 161106 (2018)

[3] Ding, Lei, et al. "Large spin-driven dielectric response and magnetoelectric coupling in the buckled honeycomb Fe4Nb2O9." Physical Review Materials 4.8 (2020): 084403*.*

[4] Toth, S., and B. Lake. "Linear spin wave theory for single-Q incommensurate magnetic structures." Journal of Physics: Condensed Matter 27.16 (2015): 166002.

[5] Deng, Guochu, et al. "Spin dynamics and magnetoelectric coupling mechanism of Co4 Nb2O9." Physical Review B 97.8 (2018): 085154.

[6] Dagar, Rahul, et al. "Magnetic-field-controlled multitude of spin excitation modes in magnetoelectric Co4Nb2O9 as investigated by magnetoterahertz spectroscopy." Physical Review Materials 6.7 (2022): 074409.

[7] Mehra, Brijesh Singh, et al. "Myriad of terahertz magnons with all-optical magnetoelectric functionality for efficient spin-wave computing in the honeycomb magnet Co4Ta2O9." Physical Review Applied 23.5 (2025): 054081.

[8] Narayanan, Narendirakumar, et al. "Magnetic structure and spin correlations in magnetoelectric honeycomb Mn4Ta2O9." Physical Review B 98.13 (2018): 134438.
